# Renin–aldosterone testing: The essentials

**DOI:** 10.1016/j.clinme.2026.100576

**Published:** 2026-03-31

**Authors:** Eunice K.T. Phillips, Abilash Sathyanarayanan

**Affiliations:** a Oxford University School of Medicine & Biomedical Sciences, University of Oxford, Oxford, UK; b Diabetes and Endocrinology, Nottingham University Hospitals, Nottingham, UK

**Keywords:** Primary aldosteronism, Renin, Hypertension, Screening, Hypokalaemia, Spironolactone

## Abstract

Primary aldosteronism (PA) is a common yet significantly underdiagnosed cause of secondary hypertension, estimated to affect around 9.4% of hypertensive adults. This is critical because patients with PA face markedly higher rates of adverse cardiovascular and renal outcomes, risks substantially mitigated by targeted treatment (surgery or medical therapy). Despite this, screening rates remain critically low. This article provides a practical, evidence-based approach to PA screening specifically for generalist settings, focusing on contemporary test interpretation and mitigating common pitfalls. Screening involves a simple blood test for plasma aldosterone and renin. The diagnostic hallmark is renin-independent aldosterone production, demonstrated by suppressed renin. While most antihypertensives interfere, the initial approach is to test patients on their current regimen. If results are inconclusive, medication withdrawal is warranted. Specialist referral is necessary when results are suggestive of PA, or in complex cases like chronic kidney disease. Improving PA screening offers a significant opportunity for substantial global benefit in reducing hypertension-related morbidity and mortality.

## Highlights

• Primary Aldosteronism (PA) is a significantly underdiagnosed and common cause of secondary hypertension.
• PA leads to higher rates of adverse cardiovascular and renal events compared to essential hypertension.
• Screening is straightforward using an outpatient blood test for plasma aldosterone and renin.
• Most first line antihypertensives can interfere with testing, usually increasing the risk of a false negative.
• PA screening and effective treatment offer a high-impact way to cut global hypertension-related deaths.

## Introduction

Primary aldosteronism (PA) – an adrenal disorder of inappropriate and autonomous aldosterone excess – is a common yet underdiagnosed and under-treated cause of secondary hypertension. Once considered rare, PA is now estimated to have a global prevalence of around 9.4%, with an even greater prevalence in patients with hypertension with additional characteristics such as younger age, male sex and hypokalaemia.^1^ Given that at least 1.4 billion adults are estimated to have hypertension worldwide,^2^ even a conservative estimate suggests that at least 100 million adults may be living with PA – many likely to be undiagnosed and thus untreated.

This carries major implications, for individual patient care and public health, for four reasons. The first is the high prevalence. Secondly, patients with PA experience markedly higher rates of adverse cardiovascular and renal outcomes compared to those with essential hypertension.^3^ Thirdly, conventional antihypertensive regimens are often insufficient, whereas targeted treatments – such as an adrenalectomy in unilateral PA or the use of mineralocorticoid receptor antagonists (MRAs) in bilateral PA – substantially improve outcomes.^4^ Finally, screening for PA can be done in the generalist setting with a simple outpatient blood test.^5^

Despite this, PA remains heavily underdiagnosed. Real-world data suggest that even in high-risk hypertensive patients in well-resourced settings, fewer than 5% of eligible hypertensive patients are screened for PA. Contributing factors are thought to include limited clinician awareness and the perceived complexity of test interpretation.^6^

Encouragingly, improved awareness, screening and management offer a significant opportunity for substantial global benefit with comparatively modest effort. Given what stands to be gained, this article aims to provide a practical approach to screening for PA in the generalist/primary care setting based on contemporary ideas of test interpretation. It focuses on the common pitfalls and provides guidance on when specialist involvement is likely to be beneficial.

### Principles of testing

The recommended screening test for PA is serum/plasma aldosterone and the plasma renin (obtained alongside plasma potassium). The renin measurement may be in the form of a renin concentration or activity. The values that indicate the presence of PA vary based on the test used, therefore, clinicians should familiarise themselves with their local assay characteristics.^7^

The pretest probability of PA among patients with hypertension is much higher than thought previously. Therefore, in testing for a condition in a population with high prevalence, it is essential that false negatives are minimised – ie that patients with PA are not missed while screening. This necessitates using a screening test with a high sensitivity and ensuring optimal preanalytical factors to maximise the sensitivity of the test.^8^

Renin and aldosterone levels are best obtained in the morning, about 2 h after waking up. Sitting and resting for 15 min is recommended prior to the test.^9^ Patients should avoid a low salt diet for 3 days prior to the test.^10^ In women, the test is best performed 7 days after the first day of the last period.^11^ Although all the above factors increase the sensitivity of the test, a random/inpatient sample can also be interpreted, especially if the test is positive.

There is significant intraindividual variability of aldosterone and renin values. The sensitivity of a single test using the aldosterone : renin ratio (ARR) for interpretation is around 70%, but can be as low as 25% if more restrictive ARR cut-off values are employed. Therefore, at least two tests are recommended before excluding the possibility of PA in a patient.^12^

### Interpretation

Conventionally, the ARR was primarily used in the interpretation of the test. It is still widely used in many guidelines. However, contemporary guidelines recommend that the aldosterone and renin values are best interpreted individually.^4^ This also allows interpretation when there are factors that influence the aldosterone and renin values. The first step in interpretation is to establish the renin status. The hallmark of PA is ‘renin-independent’ aldosterone production. This is demonstrated by the presence of low renin on the blood test; and this signifies the existence of a PA pathophysiology in a patient (this is generally a plasma renin activity <1.0 ng/mL/h or a direct renin concentration <8.2 mU/L).^4^, ^7^ Once low renin is identified, the next step is to look at the aldosterone level to see if it exceeds the local assay cut-off to determine if PA can be diagnosed.

### Medication interference

Most first-line antihypertensive medications interfere with screening for PA. Most antihypertensive medications increase the chance of a false negative (by either increasing renin levels, decreasing aldosterone levels, or both).

If patients are already on medications that interfere with testing, an approach that is recommended by the guidelines is to do the aldosterone and renin testing without altering the medications. If this demonstrates results suggestive of PA (ie low renin), then this is likely to be a true positive and warrants referral to a specialist.^8^ If the result is not in keeping with PA, then given the reduced sensitivity of the test in this situation, medication withdrawal and reassessment are usually warranted. Doxazosin, prazosin, modified release verapamil, hydralazine and moxonidine are some of the antihypertensive agents that have minimal effects on the renin and aldosterone and therefore can be used during interfering medication withdrawal if required.

Beta-blockers and central alpha-2 agonists (clonidine, methyldopa) can lead to false positive results (by lowering renin). Therefore, if the testing is not in keeping with PA while patients are only on these drugs, then it is unlikely to be a false negative.^4^, ^5^

Due to the above issues, the best window of opportunity in testing for PA is when a patient is initially diagnosed with hypertension, prior to commencing first-line treatment. In some patients, it may not be possible to safely withdraw or transition them to non-interfering agents. These patients should be discussed with a specialist.

Non-antihypertensive medications that interfere with testing that can be overlooked are – oral contraceptive pills (especially drospirenone, which has anti-mineralocorticoid activity), non-steroidal anti-inflammatory agents, glucocorticoids, antidepressants, sodium-glucose co-transporter-2 inhibitors and sympathomimetics (eg pseudoephedrine).^4^, ^5^

### Effect of potassium

Serum potassium is a potent stimulator of aldosterone production, independent of renin levels. Therefore, hypokalaemia can lead to a false negative result.^13^ Traditionally, the recommendation has been to perform testing for PA after correcting any hypokalaemia. However, testing can be done when the patient has hypokalaemia, and if an elevated aldosterone and suppressed renin are demonstrated despite hypokalaemia, then PA is extremely likely. Even if an overtly elevated aldosterone is not demonstrated, these patients with hypokalaemia and suppressed renin warrant specialist involvement. In addition, hypokalaemia is only present in a minority of patients with PA, and lack of hypokalaemia should not preclude testing for PA.^4^

## Chronic kidney disease

Although the exact prevalence of PA in patients with chronic kidney disease (CKD) is unknown, presence of PA is associated with an increased risk of chronic kidney disease.^1^ In addition, the presence of PA masks the severity of renal disease by leading to an overestimation of the eGFR. The overestimation of eGFR in PA occurs because high aldosterone levels induce glomerular hyperfiltration, which can mask underlying renal impairment until the PA is treated and the ‘masking’ effect is removed. Treating PA (medical or surgical) in the context of CKD is associated with improved renal outcomes. However, screening and further work up for PA in the presence of CKD can be complicated.^14^ CKD is also associated with both high (via decreased renal perfusion) and low (via volume expansion or renal tissue loss) renin levels, depending on the aetiology and the stage of the CKD.^4^, ^15^ Therefore, in patients with CKD, generalists should have a high suspicion of PA and liaise with specialists early in the disease process, as the interpretation of renin and aldosterone may be complicated.

## Low-renin hypertension

Many patients with hypertension may demonstrate a low renin, but many of these patients will not have high aldosterone levels to suggest PA. Some of these patients may have a forme fruste type of PA, but other patients may have an alternative explanation for the low renin (eg cortisol excess, Liddle syndrome). Therefore, if low-renin hypertension is identified, involvement of specialist care can be helpful in making long-term treatment decisions. There is also evidence that the subgroup of patients with a PA pathophysiology to explain their low-renin state may benefit from treatment with MRAs over the standard first-line antihypertensives.^16^ In addition, Black patients have a higher prevalence of the low-renin phenotype, and the heterogeneity of the treatment effects in this population is unclear.^17^

## Conclusion

Guidelines vary in their recommendation on who should be screened for PA (universal screening in all hypertensive patients versus targeted screening in specific subsets) – predominantly based on local resource availability.^4^, ^5^ Data show that about 25% of all patients seen in a primary care setting warrant further investigation for PA.^18^ Spironolactone is available even in resource-poor settings and it is on the essential medications list (World Health Organization). If PA is identified, access to adrenal venous sampling and adrenalectomy may be limited in some settings and spironolactone can be considered for treatment in these patients instead.^19^ However, the lack of a diagnosis will relegate these patients to a lifetime of inadequate care and poorer outcomes. Hypertension is the leading global cause of adverse cardiovascular outcomes and premature death – and most of this is preventable.^2^ In reducing human suffering and death from hypertension worldwide, an appropriate diagnosis is not optional.

## CRediT authorship contribution statement

**Abilash Sathyanarayanan:** Writing – review & editing, Writing – original draft, Conceptualization. **Eunice K.T. Phillips:** Writing – review & editing, Writing – original draft, Conceptualization.

## Funding

This article did not receive any specific grant from funding agencies in the public, commercial or not-for-profit sectors.

## Declaration of competing interest

The authors declare the following financial interests/personal relationships which may be considered as potential competing interests: A.S. is a core associate editor of *Clinical Medicine*. A.S. had no involvement in the peer review of this article and had no access to information regarding its peer review. Full responsibility for the editorial process for this article was delegated to another journal editor. If there are other authors, they declare that they have no known competing financial interests or personal relationships that could have appeared to influence the work reported in this paper.
